# Valley-optical absorption in planar transition metal dichalcogenide superlattices

**DOI:** 10.1038/s41598-023-31950-9

**Published:** 2023-04-03

**Authors:** R. Hashemi, S. Shojaei, B. Rezaei, Zheng Liu

**Affiliations:** 1 Faculty of Physics, University of Tabriz, Tabriz, Iran; 2 Research Institute for Applied Physics and Astronomy (RIAPA), University of Tabriz, Tabriz, Iran; 3 School of Materials Science and Engineering, Nanyang Technological University, Singapore, Singapore

**Keywords:** Condensed-matter physics, Electronics, photonics and device physics

## Abstract

In this study, we investigate the optical absorption of a planar superlattice comprising alternatively arranged two-dimensional Transition Metal DiChalcogenide semiconductors. Within a semi-classical model and using the Dirac-like equation in the presence of light interaction as a perturbation, we obtained the governing Hamiltonian. Using this Hamiltonian, we derived a fully analytical relationship for the absorption coefficient of the structure. By calculating the effective mass for different bands and using the Drude-Lorentz model, our approach is able to determine the oscillator strength and the effective refractive index of the structure. We found that the spin–orbit coupling has important effect on the absorption coefficient and energy bands where it reduces the absorption coefficient of the structure from typical value of $11.54 ({10}^{5}{\mathrm{cm}}^{-1})$–$5.937({10}^{5}{\mathrm{cm}}^{-1})$, also the valence band experiences a significant blue shift, while the conduction band shows minor changes due to spin orbit coupling. Moreover, the role of incident light angle and light polarization were studied in details at different valleys of $K$ and ${K}^{^{\prime}}$. The most important finding is that by changing the polarization of incident light, it is possible to increase the absorption coefficients of $K$ and ${K}^{^{\prime}}$ valleys by up to 30 times. For light propagation direction close to perpendicular to the plane of the superlattice, the right-circular polarization is absorbed only by $K$ valley in contrast to the left-circular polarization, which is absorbed by the ${K}^{^{\prime}}$ valley. Our model might be used to design newly developed 2D optovalleytronic devices.

Two-dimensional (2D) materials with atomic thickness such as graphene, transition metal dichalcogenides (TMDCs), boron nitride, phosphorene, etc. are considered as highly promising materials for the next generation of electronics and photonics due to their unique electronic and optical properties^1^. Compared to conventional three-dimensional (3D) photonic materials such as GaAs and Si, 2D ones exhibit many unique properties for some important reasons. Due to quantum confinement in 2D materials, they exhibit new electronic and optical properties, which is completely different from their bulk properties. In addition, the surface of these materials naturally do not have dangling bonds; this feature leads to the possibility of integrating 2D materials with photonic systems. On the other hand, despite the low thickness, most of these materials interact strongly with light, and finally since the 2D materials cover a very wide range of electromagnetic spectrum, they can have a variety of photonics applications.

One of the most important members of the family of 2D materials is the 2DTMDC semiconductors. These 2D semiconductors represent a new class of materials, which proposed in the field of semiconductor physics and nanoscience as well as electro-optical applications. Studies on monolayer TMDCs (ML-TMDCs) have been inspired and benefited almost from the focused research and development on graphene-based systems. Studies on ML-TMDCs, as part of "beyond graphene research" in order to clarify their essential properties and new potential performance are mostly at the basic research level^2–7^. These materials have the chemical formula MX2, where M represents the transition metal atom (molybdenum, tungsten, etc.) and X represents the chalcogen atom (sulfide, selenide, telluride, etc.). In addition to having a direct band gap due to the structure inversion asymmetry, TMDCs provide access to a new freedom degree of carrier, i.e., the valley index, which leads to a new branch of physics, called valleytronics. Besides, the strong spin–orbit coupling (SOC) leads to spin–orbit splitting and provides the electron spin control by regulating the excitation energy of the laser photon^8,9^. All mentioned properties make these materials to be different from the graphene; so that TMDCs nanomaterials such as MoSe2, MoS2, WSe2 and WS2 are emerging as a new generation of photonic nanomaterials^10,11^.

One of the main challenges in the field of 2D materials is the theoretical and analytical study of their optical properties. Optical absorption in ML-TMDCs with a thickness of less than 1 nm in the visible spectral region (E = 1.590–3.263 eV) and near infrared (E = 0.827–1.590 eV) happens through a direct transition between the valence and conduction bands. Due to the strong light-matter interaction and the desirable band gap of 1 to 2 eV, the optical absorption of these materials is in the order of 5 to 10%, which is greater than the absorption of GaAs and Si^12^. The heterostructures could be used to enhance light absorption and, consequently, the development of photovoltaic systems due to various optical properties^11^.

The 2D materials, with a few nanometer in size, must eventually defeat the silicon-based devices. Creating the thinnest nanometer channels in 2D materials can be a major step towards the development of sub-nanometer electro-optical devices. However, the production of useful electronic devices requires the integration of several 2D materials into one plane, which is a very difficult challenge. Creating 2D material heterostructures enable us to engineer bands on a 2D plane and open up a new domain in materials science, physical devices and their engineering. On the other hand, a periodic structure composed of two layers with different characteristics (superlattice) has promised improving the performance of these devices, because the periodicity of the superlattice leads to improved electrical charge conduction and its control along the superlattice. Incorporation of these two concepts offer to double the performance of devices, including infrared optical detectors and solar cells, and a way to extend fully 2D transistors. Effective usage of solar energy requires adapting the performance of these structures to solar radiation. In order to enhance the functionality of these structures (absorbers) and reduce their fabrication cost, the thickness of these materials must be reduced. On the other hand, it is clear that the most interesting physical phenomenon take place at the interface of two layers, therefore, the performance of superlattice-based solar cells will be improved. Until 2015, no multiple heterostructures or 2D lateral superlattices of less than 5 nm wide, a regime in which quantum-size effects are appeared, have been reported and leaving a major challenge for 2D material research^13^. Recently, different mechanisms have been applied for investigation the optical and electronic properties of TMDC-PSL structures. In 2018, Han and et al. created superlattices, especially the planar ones with nanometer channels consisting of ML-WSe_2_ and ML-MoS_2_^14^. In 2D WSe2 and MoS2 crystals, the constituent elements are alternatively arranged in a hexagon configuration to produce a honeycomb pattern. The researchers were able to use a special technique in TMDC planar superlattices (TMDC-PSLs). However, a theoretical explanation of the molecular mechanism and an analytical study of the fundamentals underlying the formation of these nanochannels, promise that their formation can be controlled to provide the possibility of obtaining the electro-optical devices at the atomic scale based on planar heterostructure or superlattice. Zhao et al. studied theoretically the tunable electronic structure of TMDC-PSL made of WSe_2_ and MoS_2_ bilayers^15^, where the tunability of direct and indirect band gaps of the structure has been investigated using superlattice parameters through density functional theory (DFT) simulation method. In addition, the role of SOC on electronic band structure and the absorption coefficient was investigated and the absorption coefficient in the order of ${10}^{2}{\mathrm{cm}}^{-1}$ reported. In recent years, some experimental works have been made and reported on engineering the electronic band structure of TMDC-PSLs^16–21^.

In this work, we perform a comprehensive analytical investigation of the optical properties of the TMDC-PSL based on the effective Hamiltonian approach. The optical absorption coefficient and the photoluminescence intensity of the TMDC-PSL is one of the main directions of this research. In the first part, based on some reported experimental and theoretical works, a model was proposed for Hamiltonian of the TMDC-PSL structures. In the second part, using this Hamiltonian approach, we derived a fully analytical relationship for the absorption coefficient of the structure by considering the freedom degree of the valleys as well as the incident light with different polarizations. In the third part, the optical characteristics of the structure is reported by considering the effective mass, oscillator strength and effective refractive index. The effect of geometrical parameters of the superlattice on its optical absorption in different valleys discussed in details. Finally, we will summarize our main findings.

## Model and method

### TMDC-PSL Hamiltonian

**Figure 1:**
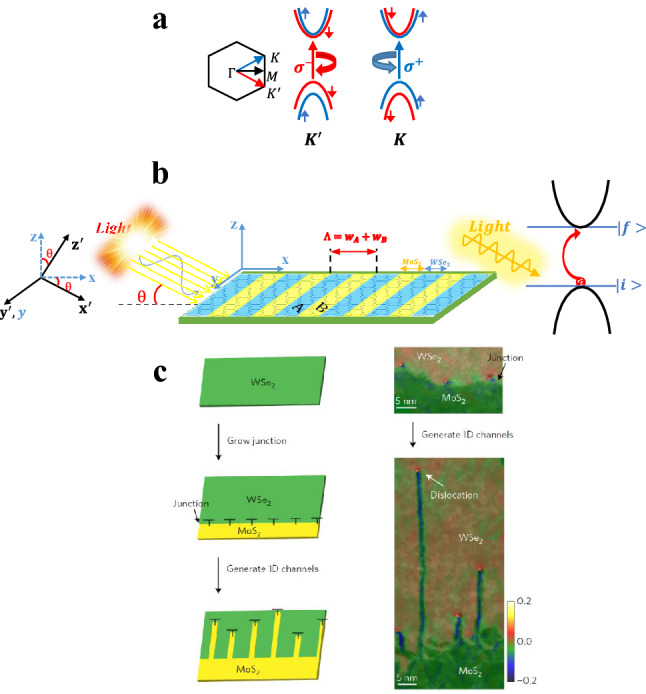
(**a**) Comparison of electronic structure in $K$ and ${K}^{^{\prime}}$ valleys for TMDC-PSL; (**b**) Schematic diagram of the impinging light on TMDC-PSL and interband transition. A, B indicate two ML-TMDCs; MoS_2_ and WSe_2_, respectively. (**c**) Formation of 1D channels**.** Left column, Schematic of the patterning process guided by misfit dislocations (marked as ‘T’) at the MoS_2_–WSe_2_ lateral heterojunction. Right column Atomic-resolution ADF-STEM images overlaid with their ${\varepsilon }_{xx}$ strain maps identifying the periodic dislocations at the interface of MoS_2_ and WSe_2_ and the 1D channels created by chemically driven migration of the interfacial dislocations as additional S and Mo atoms are added (**c**)^13^.

In our model, we consider the TMDC-PSL as shown in Fig. 1, which is formed by alternatively arranged ${MoS}_{2}/{WSe}_{2}$ in a plane labeled as A and B. This model is evidenced by the experiments for demonstration of 2D lateral multiheterojunctions and superlattices by growth of sub–2-nm quantum-well arrays in semiconductor monolayers, driven by the climb of misfit dislocations in a lattice-mismatched sulfide/selenide heterointerface^13^. We start with the ML-TMDCs Hamiltonian to obtain the band structure and transmission spectrum of TMDC-PSL^22,23^ as:

1 $$\widehat{{\text{H}}}_{i} = a_{i} t_{i} \left( {\tau _{i} k_{x} \hat{\sigma }_{x} + k_{y} \hat{\sigma }_{y} } \right) \otimes \widehat{{\mathbb{I}}} + \frac{{\Delta _{i} }}{2}\hat{\sigma }_{z} \otimes \widehat{{\mathbb{I}}} - \frac{{\lambda _{i} \tau _{i} }}{2}\left( {\hat{\sigma }_{z} - \widehat{{\mathbb{I}}}} \right) \otimes \hat{S}_{z}$$

where $i$ corresponds to the different layers of the superlattice. With definition Fermi velocity as ${v}_{F.i}=\frac{{a}_{i}{t}_{i}}{\mathrm{\hslash }}$ and SOC term as ${V}_{i}={\uplambda }_{i}{s}_{i}$, the ML-TMDCs Hamiltonian reads:

2 $$\hat{\rm H}_{i} = - i\hbar v_{F.i} \left( {\tau_{i} \sigma_{x} \partial_{x} + \sigma_{y} \partial_{y} } \right) + \frac{{\Delta_{i} }}{2}\hat{\sigma }_{z} - \tau_{i} V_{i} \frac{{\hat{\sigma }_{z} - \hat{1}}}{2}$$

The Hamiltonian of the ML-TMDC at two $K$ and ${K}^{\mathrm{^{\prime}}}$ valleys will be in the form of a $4\times 4$ matrix. Since we have ignored the coupling between the valleys, so we can express the Hamiltonian of these two valleys separately as a $2\times 2$ matrices. In our previous work, we described the analytical approach with detailed explanations for solving the governing Hamiltonian with boundary conditions to obtain the wave functions and band structure of TMDC-PSL^23^. The central symmetry breakdown caused by the SOC in ML-TMDCs leads to the valance bands splitting. These separated sub-bands have up- and down-spin characteristics (see Fig. 1a). The lack of spin degeneracy together with time reversal symmetry is the reason of the intrinsic coupling between valley and spin degrees of freedom in valence sub-bands of ML-TMDCs. Therefore, interband transitions at two valleys are allowed only for optical excitations with opposite circular polarization. In the other words, $K$ and ${K}^{\mathrm{^{\prime}}}$ valleys are excited only by right -circular (${\widehat{e}}_{RHS}$) and left -circular (${\widehat{e}}_{LHS}$) polarizations, respectively (Fig. 1). These principles are not established for the bilayer configurations due to inversion symmetry and consequently the spin degeneracy in the valleys^24^.

According to the Sturm–Liouville theorem as well as the hermeticity of the superlattice Hamiltonian^25^, we define the Hamiltonian for the whole structure as:

3 $$\hat{\rm H}_{SL} = - i\hbar \left[ {\sqrt {v_{F} \left( x \right)} \sigma_{x} \partial_{x} \sqrt {v_{F} \left( x \right)} + v_{F} \left( x \right)\sigma_{y} \partial_{y} } \right] + \frac{\Delta \left( x \right)}{2}\hat{\sigma }_{z} - V\left( x \right)\frac{{\hat{\sigma }_{z} - \hat{1}}}{2},$$

where $v_{F} \left( x \right)$, ${\Delta }\left( x \right)$, $V\left( x \right)$ and ${\uptau }\left( x \right)$ are defined as:

4 $$\begin{array}{*{20}c} {v_{F} \left( x \right) = v_{F.1} , \Delta \left( x \right) = \Delta_{1} , V\left( x \right) = V_{1} , \tau \left( x \right) = \pm 1} & {for\;A\;Layer\;region} \\ {v_{F} \left( x \right) = v_{F.2} , \Delta \left( x \right) = \Delta_{2} , V\left( x \right) = V_{2} , \tau \left( x \right) = \pm 1 } & {for\;B\;Layer\;region} \\ \end{array}$$

In the presence of an electromagnetic field the momentum is ${\varvec{p}}+\frac{e}{c}{{\varvec{A}}}_{{\varvec{o}}{\varvec{p}}{\varvec{t}}}$, where $\mathbf{p}$ and ${{\varvec{A}}}_{{\varvec{o}}{\varvec{p}}{\varvec{t}}}$ are the electron momentum operator and the vector potential, respectively. Consequently, the planar superlattice's Hamiltonian when it interacts with an electromagnetic field, can be expressed as:

5 $$\begin{array}{*{20}c} { - i\hbar \left[ {\sqrt {v_{F} \left( x \right)} \sigma_{x} \left( {\partial_{x} - \frac{e}{i\hbar c}A_{x} } \right)\sqrt {v_{F} \left( x \right)} + v_{F} \left( x \right)\sigma_{y} \left( {\partial_{y} - \frac{e}{i\hbar c}A_{y} } \right)} \right]} \\ { + \frac{\Delta \left( x \right)}{2}\hat{\sigma }_{z} - V\left( x \right)\frac{{\hat{\sigma }_{z} - \hat{1}}}{2}} \\ \end{array}$$

By considering the total Hamiltonian for a set of TMDC-PSL in the presence of electromagnetic fields in the form of ${\widehat{\mathrm{H}}}_{t}={\widehat{\mathrm{H}}}_{SL}+{\widehat{\mathrm{H}}}_{em}$, we can rewrite the above Hamiltonian as the sum of the non-interactive (${\widehat{\mathrm{H}}}_{0}$) and the interactive (${\widehat{\mathrm{H}}}_{int}$) Hamiltonians as ${\widehat{\mathrm{H}}}_{t}={\widehat{\mathrm{H}}}_{0}+{\widehat{\mathrm{H}}}_{int}$, where:

6 $$\begin{aligned} {\hat{\text{H}}}_{0} & = \left( { - {\text{i}}\hbar \left[ {\sqrt {{\text{v}}_{{\text{F}}} \left( {\text{x}} \right)} {\upsigma }_{{\text{x}}} \partial_{{\text{x}}} \sqrt {{\text{v}}_{{\text{F}}} \left( {\text{x}} \right)} + {\text{v}}_{{\text{F}}} \left( {\text{x}} \right){\upsigma }_{{\text{y}}} \partial_{{\text{y}}} } \right] + \frac{{{\Delta }\left( {\text{x}} \right)}}{2}{\hat{\sigma }}_{{\text{z}}} - {\text{V}}\left( {\text{x}} \right)\frac{{{\hat{\sigma }}_{{\text{z}}} - \hat{1}}}{2}} \right) + \left[ {{\hat{\text{H}}}_{{{\text{em}}}} } \right] \\ {\hat{\text{H}}}_{{{\text{int}}}} & = {\text{i}}\hbar \left[ {\sqrt {{\text{v}}_{{\text{F}}} \left( {\text{x}} \right)} {\upsigma }_{{\text{x}}} \frac{{\text{e}}}{{{\text{i}}\hbar {\text{c}}}}{\text{A}}_{{\text{x}}} \sqrt {{\text{v}}_{{\text{F}}} \left( {\text{x}} \right)} + {\text{v}}_{{\text{F}}} \left( {\text{x}} \right){\upsigma }_{{\text{y}}} \frac{{\text{e}}}{{{\text{i}}\hbar {\text{c}}}}{\text{A}}_{{\text{y}}} } \right], \\ \end{aligned}$$

The interaction Hamiltonian can be represented as follows by defining the relations ${\varvec{\sigma}} = \sigma_{x} {\varvec{i}} + \sigma_{y} {\varvec{j}}$ and $\user2{\sigma^{\prime}} = - \sigma_{x} {\varvec{i}} + \sigma_{y} {\varvec{j}}$ for the $K$ and $K^{\prime}$-valleys, respectively:

7 $${\hat{\text{H}}}_{int} = \left\{ {\begin{array}{*{20}c} {\frac{{ev_{F} \left( x \right)}}{c}\sigma \cdot {\varvec{A}}_{{{\varvec{opt}}}} } & {for\;K\;valley} \\ {\frac{{ev_{F} \left( x \right)}}{c}\user2{\sigma^{\prime}} \cdot {\varvec{A}}_{{{\varvec{opt}}}} } & {for \;K^{\prime}\;valley} \\ \end{array} } \right..$$

### Interaction of electromagnetic field with TMDC-PSL

Our semi-classical method often describes the properties of absorption and induction of emission, but it is not suitable for describing spontaneous emission, because the quantum nature of the optical field must be taken into account. The electromagnetic field’s action as a perturbation changes the system from its initial state ${\psi }_{i}$, to its final state ${\psi }_{f}$ , after a duration of time $t$ . We are interested in calculating the transition probability between the initial and final states, supposing that the orthonormal eigenfunctions ${\psi }_{0m}$ of the unperturbed system with Hamiltonian ${H}_{0}$ and the corresponding energies ${E}_{0m}$ are known (${H}_{0}\left(\genfrac{}{}{0pt}{}{{\psi }_{0mA}}{{\psi }_{0mB}}\right)={E}_{0m}\left(\genfrac{}{}{0pt}{}{{\psi }_{0mA}}{{\psi }_{0mB}}\right)$). Assuming that the incident light intensity is weak enough, we can omit the term ${A}^{2}$ (proportional to light intensity) in these calculations, and the system response (absorption coefficient) will be independent of the light intensity. It is also important to keep in mind that this term lacks a momentum operator, making it unable of changing the lowest-level electron state or contributing to the linear absorption of light. Therefore, the Hamiltonian's linear component is the only component that contributes to the first order perturbation theory. i.e. ${H}_{int}={H}_{A}$. Assume that ${\psi }_{0i}$ to be a set of complete and orthogonal basis, so that^23^:

8 $$\begin{aligned} \mathop \smallint \limits_{ - \infty }^{ + \infty } dq & \left( {\psi_{0l.A} \left( q \right).\psi_{0l.B} \left( q \right)} \right)^{*} \left( {\begin{array}{*{20}c} {\psi_{0m.A} \left( q \right)} \\ {\psi_{0m.B} \left( q \right)} \\ \end{array} } \right) \\ { } & = \mathop \smallint \limits_{ - \infty }^{ + \infty } dq\left[ {\psi_{0l.A}^{*} \left( q \right)\psi_{0m.A} \left( q \right) + \psi_{0l.B} \left( q \right)^{*} \left( q \right)\psi_{0m.B} \left( q \right)} \right] = \delta_{lm} \\ \end{aligned}$$

Through expansion theorem and knowing that Hamiltonian is not explicitly dependent on time, the expansion coefficients can be obtained according to the time-dependent Schrodinger equation for a two-band system, as^26^:

9 $$\alpha_{f} \left( {t_{f} } \right) = - \frac{i}{\hbar }\mathop \smallint \limits_{{t_{i} }}^{{t_{f} }} H_{int,fi} e^{{ - i\frac{{\left( {E_{0i} - E_{0f} } \right)t}}{\hbar }}} dt.$$

where ${H}_{int.fi}$ is defined as ${H}_{int.fi}=<{\psi }_{0f}\left(q\right)|{{H}_{int}|\psi }_{0i}\left(q\right)>$. The transition probability between the initial state $i$ and the final state $f$ when the time varies from ${t}_{i}$ to ${t}_{f}$ is defined as ${P}_{fi}={\left|{a}_{f}({t}_{f})\right|}^{2}$. We consider the classical optical field (${\varvec{E}}$) impinging on the system as:

10 $${\varvec{E}} = - {\varvec{E}}_{0} \cos \left( {\user2{k^{\prime}} \cdot {\varvec{r}} - \omega t} \right)\hat{\user2{e}}^{^{\prime}} = - \frac{{{\varvec{E}}_{0} }}{2}\left[ {e^{{i\left( {\user2{k^{\prime}} \cdot {\varvec{r}} - \omega t} \right)}} + e^{{ - i\left( {\user2{k^{\prime}} \cdot {\varvec{r}} - \omega t} \right)}} } \right]\hat{\user2{e}}^{^{\prime}} .$$

The ${\widehat{{\varvec{e}}}}^{\mathrm{^{\prime}}}$ is the polarization vector of the incident electromagnetic field and ω is the frequency corresponds to that field. We show the electromagnetic field in the primed coordinate system, when it impinges with an incident angle $\theta$ on the TMDC-PSL plane. Therefore, for propagation of the electromagnetic field along the direction ${\widehat{{\varvec{x}}}}^{\mathrm{^{\prime}}}$ (${{\varvec{k}}}^{\mathrm{^{\prime}}}=k{\widehat{{\varvec{x}}}}^{\mathrm{^{\prime}}}$), the circular polarization vector in the primed coordinate can be written as:

11 $$\begin{array}{*{20}l} {\hat{\user2{e}}^{^{\prime}}_{{{\varvec{RHS}}}} = \frac{{\left( {\hat{\user2{y}}^{^{\prime}} + \user2{i\hat{z}}^{^{\prime}} } \right)}}{\sqrt 2 } } \hfill & {Right - handed circular polarization vector} \hfill \\ {\hat{\user2{e}}^{^{\prime}}_{{{\varvec{LHS}}}} = \frac{{\left( {\hat{\user2{y}}^{^{\prime}} - \user2{i\hat{z}}^{^{\prime}} } \right)}}{\sqrt 2 }} \hfill & {Left - handed circular polarization vector} \hfill \\ \end{array}$$

By defining the vector potential as ${\varvec{A}}_{{{\varvec{opt}}}} = \frac{{ic{\varvec{E}}}}{\omega }$, the interaction Hamiltonian for $K$ –valley :

12 $${\hat{\text{H}}}_{int} = - \frac{{ev_{F} \left( x \right)}}{i\omega }{\varvec{\sigma}} \cdot {\varvec{E}} = \frac{{E_{0} ev_{F} \left( x \right)}}{2i\omega }\left[ {e^{{i\left( {\user2{k^{\prime}} \cdot {\varvec{r}} - \omega t} \right)}} + e^{{ - i\left( {\user2{k^{\prime}} \cdot {\varvec{r}} - \omega t} \right)}} } \right]{\varvec{\sigma}} \cdot \hat{\user2{e}}^{\user2{^{\prime}}}$$

It is clear that when light radiates to the system, it may induce electric dipole and electric quadrupole, as well as magnetic dipole in the system. Using the above equation we can write the interaction Hamiltonian as ${\widehat{\mathrm{H}}}_{int}\propto {e}^{i\left({\varvec{k}}\cdot {\varvec{r}}\right)}{\varvec{p}}$**.** Assuming that the incident wavelength ($\lambda$) is much larger than the TMDC-PSL unit cell ($\lambda \gg \Lambda \sim 3-10 \mathrm{nm}$), so it will experiences the structure as a homogeneous environment with a definite band structure. We can expand ${\widehat{\mathrm{H}}}_{int}$ as ${e}^{i\left({\varvec{k}}\cdot {\varvec{r}}\right)}{\varvec{p}}\cong {\varvec{p}}+i\left({\varvec{k}}\cdot {\varvec{r}}\right){\varvec{p}}\cong {\varvec{p}}$, where the ${\varvec{p}}$ term is corresponds to electric dipole transitions and the $i\left({\varvec{k}}\cdot {\varvec{r}}\right){\varvec{p}}$ term is related to electric quadrupole and magnetic dipole transitions. The last term is often less important in the visible and infrared spectrum. However, quadrupole transitions play a significant role in the far UV spectrum. As a result, using the electric dipole approximation at long wavelengths, the ${\widehat{\mathrm{H}}}_{int}$ can be obtained as follows:

13 $${\hat{\text{H}}}_{int} = \frac{{E_{0} ev_{F} \left( x \right)}}{2i\omega }\left[ {e^{i\omega t} + e^{ - i\omega t} } \right]{\varvec{\sigma}} \cdot \hat{\user2{e}}^{\user2{^{\prime}}}$$

To obtain ${P}_{fi}$, one must calculate ${\int }_{{t}_{i}}^{{t}_{f}}{H}_{int.fi}{e}^{-i\left({E}_{0i}-{E}_{0f}\right)t/\hslash}dt$. Using the rotating wave approximation and assumption that ${E}_{0f}-{E}_{0i}=\hslash {\omega }_{fi}$ and $\left(x\right)=<{\psi }_{0f}\left(q\right)\left|{v}_{F}\left(x\right){\varvec{\sigma}}\cdot {\widehat{{\varvec{e}}}}^{\boldsymbol{^{\prime}}}\right|{\psi }_{0i}\left(q\right)>$ , and the wavefunctions do not explicitly depend on time the integral of Eq. (9) is as follows:

14 $$\mathop \smallint \limits_{{t_{i} }}^{{t_{f} }} H_{int.fi} e^{{{\raise0.7ex\hbox{${ - i\left( {E_{0i} - E_{0f} } \right)t}$} \!\mathord{\left/ {\vphantom {{ - i\left( {E_{0i} - E_{0f} } \right)t} \hbar }}\right.\kern-0pt} \!\lower0.7ex\hbox{$\hbar $}}}} dt = \frac{{E_{0} eF\left( x \right)}}{i\omega }\left[ {{\text{e}}^{{i\frac{{\left( {\omega_{fi} - \omega } \right)t}}{2}}} \frac{{{\text{sin}}\left[ {i\frac{{\left( {\omega_{fi} - \omega } \right)t}}{2}} \right]}}{{\left( {\omega_{fi} - \omega } \right)}}} \right]$$

Therefore, using the Eq. (14), and definition of the delta function in the form of $${\updelta }\left( x \right) = \mathop {\lim }\limits_{ \in \to 0} \frac{}{\pi }\frac{{{\text{sin}}^{2} {\raise0.7ex\hbox{$x$} \!\mathord{\left/ {\vphantom {x }}\right.\kern-0pt} \!\lower0.7ex\hbox{$$}}}}{{x^{2} }}$$, we reach the final relation for the transition probability as:

15 $$P_{fi} = \left| {\frac{{E_{0} eF\left( x \right)}}{\hbar \omega }} \right|^{2} \frac{\pi t}{2}\delta \left( {\omega_{fi} - \omega } \right)$$

where the $\updelta \left({\omega }_{fi}-\omega \right)$ function is related to the energy conservation law for the optically driven interband transition.

### Interband transition

The interband absorption is one of the most important mechanisms for studying the optical and electronic properties of direct bad gap systems. The dependence of absorption on the frequency of electromagnetic radiation can be calculated using the absorption coefficient relation. If the structure is nondegenerate, and not strongly doped, where the valence band is practically fully occupied and the conduction band is empty, the occupation probability of the bands need not be explicitly taken into account, and we can think of ${H}_{int.fi}$ as changing slowly on the constant energy surface. Then we will have the probability of transition in the form of ${P}_{tot}={\left|\frac{{E}_{0}eF\left(x\right)}{\mathrm{\hslash }\omega }\right|}^{2}\frac{t}{8\pi }{\int }_{-\infty }^{+\infty }\updelta \left({\omega }_{fi}-\omega \right)d{\varvec{K}}$. If we use the divergence theorem and consider the element of integration in Eq. (9) as a surface for 2D structure, we get^26^:

16 $$P_{tot} = \left| {\frac{{E_{0} eF\left( x \right)}}{\hbar \omega }} \right|^{2} \frac{t}{8\pi }\hbar \smallint \frac{ds}{{\left| {\nabla_{k} \left( {E_{f} - E_{i} } \right)} \right|_{{E_{f} - E_{i} = \hbar \omega }} }}.$$

where $S$ is the surface of energy and ${{E}_{f}-E}_{i}=\mathrm{\hslash }\omega$. In addition, if the valence and conduction bands are both homogeneous and parabolic with an extremum at ${\varvec{K}}=0$^23^, the valence and the conduction band energies are defined as ${E}_{v}=-\frac{{\mathrm{\hslash }}^{2}{K}^{2}}{2{m}_{v}}$ and ${E}_{c}={E}_{g}+\frac{{\mathrm{\hslash }}^{2}{K}^{2}}{2{m}_{c}}$, respectively, where ${{E}_{c}-E}_{v}=\mathrm{\hslash }\omega$ holds for optical transition. In this regard, because the transition is close to the maximum and minimum points of the Brillouin zone (${\varvec{K}}=0$), the parabolic or effective mass approximation is valid. The transition probability for this planar superlattice reads:

17 $$P_{tot} = \frac{1}{2\sqrt 2 }\left| {E_{0} eF\left( x \right)} \right|^{2} \left( {\frac{{\left( {m_{r} } \right)^{{{\raise0.7ex\hbox{$3$} \!\mathord{\left/ {\vphantom {3 2}}\right.\kern-0pt} \!\lower0.7ex\hbox{$2$}}}} }}{{\hbar^{2} }}} \right)\left( {\frac{{\sqrt {\hbar \omega - E_{g} } }}{{\left( {\hbar \omega } \right)^{2} }}} \right)t$$

As the reduced mass is defined as ${m}_{r}=\frac{{m}_{c}{m}_{v}}{{m}_{c}+{m}_{v}}$.

### Transition probability and absorption coefficient

The absorption coefficient $\mathrm{\alpha }$ is defined as the transition rate per unit quantum flux ($\mathrm{\varnothing }$). Quantum flux is also defined as the number of incident photons per unit area per unit time and can be calculated from the time average of the Poynting vector S ($<{\varvec{S}}>=\frac{1}{2}{c}_{0}{\varepsilon }_{0}n{|{E}_{0}|}^{2}$) per incident energy unit ($\mathrm{\varnothing }=\frac{<{\varvec{S}}>}{\mathrm{\hslash }\omega }$). Therefore, the relationship between the absorption coefficient and the Quantum flux is defined as $\mathrm{\alpha }=\left(\frac{1}{\mathrm{\varnothing }}\right)\frac{{P}_{tot}}{t}=\left(\frac{2\mathrm{\hslash }\omega }{{c}_{0}{\varepsilon }_{0}n{\left|{E}_{0}\right|}^{2}}\right)\left(\frac{{P}_{tot}}{t}\right)$. By placing ${P}_{tot}$ using the Eq. (17), a fully analytical relation for the absorption coefficient of the TMDC-PSL is obtained as:

18 $${\upalpha } = \left( {\frac{{e^{2} \left( {m_{r} } \right)^{{{\raise0.7ex\hbox{$3$} \!\mathord{\left/ {\vphantom {3 2}}\right.\kern-0pt} \!\lower0.7ex\hbox{$2$}}}} }}{{\sqrt 2 c_{0} \varepsilon_{0} n\hbar^{2} }}} \right)\left| {F\left( x \right)} \right|^{2} \left[ {\left( {\frac{{\sqrt {\hbar \omega - E_{g} } }}{\hbar \omega }} \right)} \right]{\Theta }\left( {\hbar \omega - E_{g} } \right)$$

where $\Theta \left(\mathrm{\hslash }\omega -{E}_{g}\right)$ is the step function and indicates the conservation of energy in optical transition. As it was discussed before, the up (down) spin corresponds to $K$(${K}^{\mathrm{^{\prime}}}$) valley and the parameter $F (x)$ can be attributed to $K$ and ${K}^{\mathrm{^{\prime}}}$ valley, separately, considering that the incident light polarizations are ${{\widehat{{\varvec{e}}}}^{\mathrm{^{\prime}}}}_{{\varvec{R}}{\varvec{H}}{\varvec{S}}}$ and ${{\widehat{{\varvec{e}}}}^{\mathrm{^{\prime}}}}_{{\varvec{L}}{\varvec{H}}{\varvec{S}}}$. Additionally, as seen in Fig. 1, light interacts with the periodicity of the structure along the x-axis at incident angle $\theta$. Hence, from the geometry of the system, it is necessary that ${x}^{\mathrm{^{\prime}}}$ and ${z}^{\mathrm{^{\prime}}}$ axes rotate around the y-axis by the angle θ through the rotation matrix transformation $${\mathrm{R}}_{y}\left(\theta \right)=\left(\begin{array}{ccc}\mathrm{cos}\theta & 0& \mathrm{sin}\theta \\ 0& 1& 0\\ -\mathrm{sin}\theta & 0& \mathrm{cos}\theta \end{array}\right)$$ that converts to the prime coordinates. Finally, after detailed calculations and considering the definitions ${F}_{1(2)}\left(x\right)={\int }_{-\infty }^{+\infty }dx{v}_{F}\left(x\right){g}_{1(2)}$ and ${g}_{1(2)}=\left[{{\psi }_{0f.A}}^{*}\left(q\right){\psi }_{0i.B}\left(q\right)\right]\pm \left[{{\psi }_{0f.B}}^{*}\left(q\right){\psi }_{0i.A}\left(q\right)\right]$, we get the following relation:

19 $$\begin{aligned} F\left( x \right) & = \left\langle {\psi_{0f} \left( q \right)\left| {v_{F} \left( x \right){\varvec{\sigma}} \cdot \hat{\user2{e}}^{^{\prime}} } \right|\psi_{0i} \left( q \right)} \right\rangle { } \\ { } & = \left\{ {\begin{array}{*{20}c} {F_{RHS} \left( x \right) = \frac{1}{\sqrt 2 i}\left( { \pm \sin \theta F_{1} \left( x \right) + F_{2} \left( x \right)} \right) for K\left( {K^{\prime}} \right) valley} \\ {F_{LHS} \left( x \right) = \frac{1}{\sqrt 2 i}\left( { \mp \sin \theta F_{1} \left( x \right) + F_{2} \left( x \right)} \right) for K\left( {K^{\prime}} \right) valley} \\ \end{array} } \right. \\ \end{aligned}$$

### The effective refractive index

According to Eq. (18), the key parameter for obtaining the absorption coefficient is the effective refractive index of the TMDC-PSL. One of the most suitable methods for calculating this quantity is the Drude–Lorentz model^26^. This model describes the general behavior of the dielectric function as:

20 $$\varepsilon \left( \omega \right) = \varepsilon_{0} + \left( {\frac{{N_{0} e^{2} }}{{m_{r} L_{z} }}} \right)\mathop \sum \limits_{f} \frac{{f_{fi} }}{{\omega_{fi}^{2} - \omega^{2} - i\omega \Gamma_{fi} }}.$$

Due to the 2D structure, ${N}_{0}$ is the number of dipoles per unit area and ${L}_{z}$ is the thickness of the 2D structure. As this form of the dielectric function is valid in purely quantum description, we are able to determine the magnitude of both parameters ${f}_{fi}$ and the damping constants ${\Gamma }_{fi}$. In quantum mechanics, the environment material is modeled as a set of oscillators with different excitation frequencies, ${\omega }_{fi}=\frac{({E}_{f}-{E}_{i})}{\mathrm{\hslash }}$, which is excited from the initial state $i$ to the final state $f$ by applying an electromagnetic field. The dielectric function of the material system is obtained by summing the contribution of all oscillators, regardless of their final state. Dimensionless parameter ${f}_{fi}$ is called "oscillator strength" with frequency ${\omega }_{fi}$, which is defined as ${f}_{fi}=\left(\frac{2{m}_{r}{\omega }_{fi}}{{e}^{2}\hslash }\right){|{{\varvec{d}}}_{fi}|}^{2}$, so that ${d}_{fi}$ is the element of dipole moment matrix. Using the relationship between the dielectric function and the effective refractive index of the structure, ${\mathrm{n}}_{eff}^{2}=\frac{\varepsilon (\omega )}{{\varepsilon }_{0}}$, as well as ${n}_{eff}={n}_{re}+i{n}_{im}$, the real and imaginary parts of the ${n}_{eff}$ can be obtained as:

21 $$n_{re}^{2} = \frac{1}{{2\varepsilon_{0} }}\left[ {\sqrt {\varepsilon_{re}^{2} + \varepsilon_{im}^{2} } + \varepsilon_{re} } \right] , n_{im}^{2} = \frac{1}{{2\varepsilon_{0} }}\left[ {\sqrt {\varepsilon_{re}^{2} + \varepsilon_{im}^{2} } - \varepsilon_{re} } \right].$$

Obtaining the dipole moment ${{\varvec{d}}}_{fi}=<{\psi }_{f}\left|e{\varvec{r}}\right|{\psi }_{i}>$ for the TMDC-PSL is the heart of our calculation and model. The system's band structure and wavefunctions, which were explored in great detail in our previous study^23^, are necessary to accomplish this goal. Thus, by putting equation $\left[{\varvec{r}}.{\widehat{\mathrm{H}}}_{t}\left(x\right)\right]=i\mathrm{\hslash }{v}_{F}(x){\varvec{\upsigma}}$ within the bracket, we get:

22 $$\left\langle {\psi_{0f} \left| {\left[ {{\varvec{r}}.{\hat{\text{H}}}_{t} \left( x \right)} \right]} \right|\psi_{0i} } \right\rangle = i\hbar \left\langle {\psi_{0f} \left| {v_{F} \left( x \right){{\varvec{\upsigma}}}} \right|\psi_{0i} } \right\rangle$$

On the other hand, by rewriting the displacement relation as $\left\langle {\psi_{0f} \left| {\left[ {{\varvec{r}}.{\hat{\text{H}}}_{t} \left( x \right)} \right]} \right|\psi_{0i} } \right\rangle = \left\langle {\psi_{0f} \left| {{\varvec{r}}{\hat{\text{H}}}_{t} } \right|\psi_{0i} } \right\rangle - \left\langle {\psi_{0f} \left| {{\hat{\text{H}}}_{t} {\varvec{r}}} \right|\psi_{0i} } \right\rangle = \left( {E_{i} - E_{f} } \right)\left\langle {\psi_{0f} \left| {\varvec{r}} \right|\psi_{0i} } \right\rangle$, and by comparing it with relation (22), the dipole moment matrix is:

23 $${\varvec{d}}_{fi} = \left\langle {\psi_{0f} \left| {e{\varvec{r}}} \right|\psi_{0i} } \right\rangle = \frac{e}{{i{\upomega }_{fi} }}\left\langle {\psi_{0f} \left| {v_{F} \left( x \right){{\varvec{\upsigma}}}} \right|\psi_{0i} } \right\rangle { }$$

Therefore, the final form of the oscillator strength is obtained as $f_{fi} = \left( {\frac{2m}{{\hbar \omega_{fi} }}} \right)\left| {\left\langle {\psi_{0f} \left| {v_{F} \left( x \right){{\varvec{\upsigma}}}} \right|\psi_{0i} } \right\rangle } \right|^{2}$. Finally, by detailed calculations of $f_{fi}$ for the two $K$ and $K^{\prime}$ valleys, the final equation is obtained:

24 $$f_{fi} = f_{fi}^{^{\prime}} = \left( {\frac{2m}{{\hbar \omega_{fi} }}} \right)\left( {\left| {F_{1} \left( x \right)} \right|^{2} + \left| {F_{2} \left( x \right)} \right|^{2} } \right),$$

where ${f}_{fi}$ and ${{f}_{fi}}^{\mathrm{^{\prime}}}$ are oscillator strength for $K$ and ${K}^{^{\prime}}$ valley, respectively.

## Results and discussion

**Figure 2:**
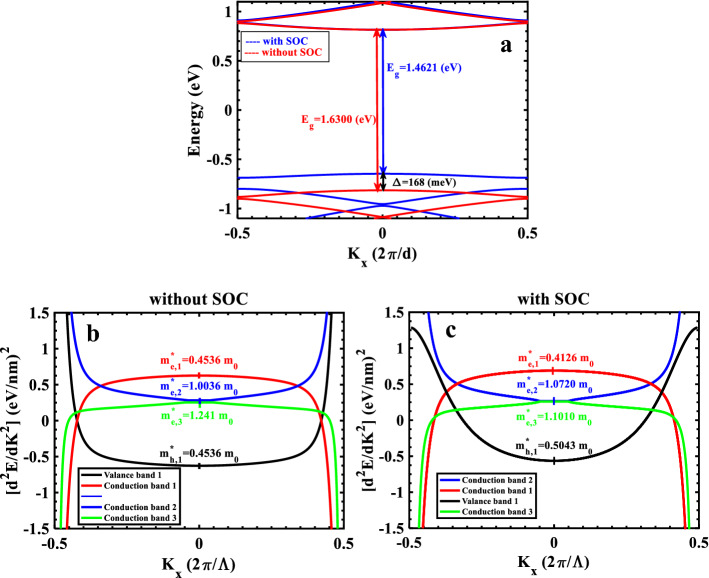
(**a**) Comparison of the band structures for two cases: with and without SOC; (**b**, **c**) the second derivative of the band structure, and the relevant effective masses around the point K = 0, without SOC and with SOC, respectively*.*

In this section we will report our findings with discussions. During the calculations, the incident angle of the light is fixed at ${\theta }_{inc}=\frac{\pi }{4}$ and the broadening factor at $\Gamma =50\mathrm{ meV}$^27^. We consider the electron density of the TMDC-PSL in the order of electron density of ML-TMDCs, ${n}_{i}=1\times {10}^{13} {\mathrm{cm}}^{-2}$, at temperature $T=300 K$^28,29^. The length of the TMDC-PSL in the direction of the periodicity equals to ${L}_{B}=50\Lambda +{w}_{A}$, where the unit cell size is $\Lambda =5({z}_{A}+{z}_{B})=3.2515\mathrm{ nm}$. ${z}_{A,B}$ is the width of the hexagons in ML-TMDCs, ${w}_{A}$ is the width of the layer A (see Ref.^17^ for details), and the thickness of the structure equals to the thickness of the ML-TMDCs, ${L}_{z}=0.646\mathrm{ nm}$. These parameters are fixed during the calculations. The study of the TMDC-optical PL's properties needs a thorough investigation of its electronic characteristics, such as the band structure and wavefunction behavior, which have previously been explored in great detail in our prior work^23^. Figure 2a, shows the electronic band structure of the TMDC-PSL for above mentioned parameters. As seen, the energy gap without SOC (red line) equals to ${\mathrm{E}}_{g}=1.630 eV$, and it equals to ${\mathrm{E}}_{g}=1.462 eV$ in the presence of SOC (blue line), where the minimum conduction band has very little displacement, and the maximum valence band experiences more displacement toward higher energies (blue shift). Therefore, the SOC has a significant effect on electronic band structure of the TMDC-PSL. Since the near-maximum valence band states are composed of ${d}_{\pm 2}$ orbitals of the transition metal atoms, they exhibit a significant spin–orbit splitting that ranges from 150 meV for $ML-Mo{S}_{2}$ to 450 meV for $ML-W{Se}_{2}$^30–38^. We found an splitting of 168 meV for TMDC-PSL made of $ML-Mo{S}_{2}$ and $ML-W{Se}_{2}$. The states near-minimum conduction band, On the other hand, are constituted of ${d}_{0}$ orbitals, which results in a considerably smaller spin–orbit splitting: Based on these two effects, it is expected that the contribution of spin signs to spin–orbit splitting will have an opposite effect, leading to the prediction of two distinctive issues. Therefore, it is expected that according to these two effects, the contribution of spin signs in spin–orbit splitting have opposite effect, hence two distinct issues can be predicted. First, if we have same spin directions in the highest valence subband and the lowest conduction subband, the optical transition is allowed for this ground state configuration, or in the other words, "bright" optical transition occurs, second, if these spins are in the opposite directions, optical transitions are not allowed and "dark" optical transitions happens. This scheme predicts two general states to participate in the transition between the valence and conduction subbands: The incident light with right-handed circular polarization (${\varvec{S}}\uparrow , {\varvec{K}}, {\widehat{{\varvec{e}}}}_{{\varvec{R}}{\varvec{H}}{\varvec{S}}}$) allows the transition between the states with up spin at $K$-valley, while the left-handed circular polarization (${\varvec{S}}\downarrow , {{\varvec{K}}}^{\mathrm{^{\prime}}}, {\widehat{{\varvec{e}}}}_{{\varvec{L}}{\varvec{H}}{\varvec{S}}}$) allows the transition between those with down spin at ${K}^{\mathrm{^{\prime}}}$-valley. In this work, we study these two special cases. It is worth to note that the study of the electronic band structure for these two states reveals exactly the same band structure as may be predicted from the behavior of TMDCs monolayers. One of the most important factors in the study of various structures is the calculation of the effective mass of carriers for different bands, because it plays a key role in the electronic and optical applications of the structure, including the efficiency of the solar cell or the speed of an integrated circuit based on our model structure. The effective masse of electrons and holes in ML-TMDCs monolayers are ${m}_{e,h}^{*}=0.32-0.86 {m}_{0}$^37^. As shown in Fig. 2a, the shape of TMDC-PSL's band structure around the point $K=0$ is parabolic, so we can calculate the effective masse of different bands using the effective mass approximation through the relation $\frac{1}{{m}^{*}}=\frac{1}{{\mathrm{\hslash }}^{2}}|\frac{{d}^{2}E}{d{K}^{2}}|$.In Fig. 2b the effective mass is reported without SOC. Due to the symmetry of the band structure, the effective mass of conduction and valence bands are equal, ${m}_{e}^{*}={m}_{h}^{*}=0.4536 {m}_{0}$. Because the effective mass depends on the bandwidth, the carriers feel less mass than the free electron mass (${m}_{0}$) in the first conduction and valence bands, but for the higher bands the effective mass of the carriers is greater than ${m}_{0}$. In the other words, , carriers strongly feel the periodic potential of the superlattice structure strongly at higher energies. In Fig. 2c, the effective mass of different bands is calculated in the presence of SOC. In this case, the effective mass of first valence (holes) and conduction (electron) bands are obtained as ${m}_{h}^{*}=0.5043 {m}_{0}$ and ${m}_{e}^{*}=0.4126 {m}_{0}$, respectively. Table 1 summarizes the effective mass of carriers for different bands in two cases (with and without SOC).

**Table 1:** Effective mass of electrons and holes for different bands with and without SOC.

*	${m}_{h,1}^{*} ({m}_{0})$	${m}_{e,1}^{*} ({m}_{0})$	${m}_{e,2}^{*} ({m}_{0})$	${m}_{e,3}^{*} ({m}_{0})$
$\mathrm{Without SOC}$	0.4536	0.4536	1.0036	1.2410
$\mathrm{With SOC}$	0.5043	0.4126	1.0720	1.1010

**Figure 3:**
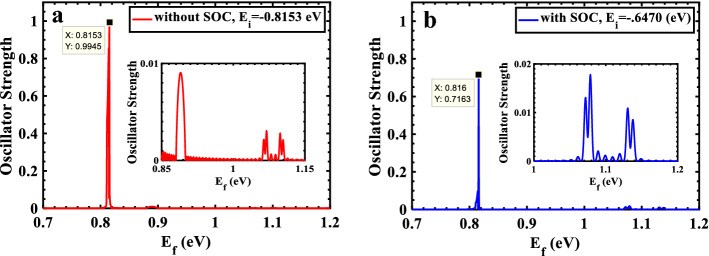
Behavior of oscillator strength for TMDC-PSL vs. different final states (**a**) without SOC and ${E}_{i}=-0.8153 eV$ and (**b**) with SOC and ${E}_{i}=-0.6470 eV$.

When an electron in the valence band is excited to the conduction band, the "oscillator strength" (OS) indicates the probability of transition of a created electrical dipole. We study the OS as a function of the final states energies (${E}_{f}$) to obtain the most desirable energy states for optimizing different optical devices. In Fig. 3a, OS is demonstrated for allowed transitions without SOC from the ground state energy ${E}_{i}=-0.8153\mathrm{ eV}$. In this figure, a very strong peak at ${E}_{f}=+0.8153 \mathrm{eV}$ is observed, indicating the transition at energy gap (transition between the valence and conduction bands extrema) and most probable optical absorption and consequently strongest photoluminescence at this energy. Additionally, Fig. 3a shows that the OS peaks for transitions at the higher band edges are substantially weaker than those at the edge of the conduction band (two orders of magnitudes). The same study was performed with SOC, as shown in Fig. 3b. Here the ground state energy is ${E}_{i} = -0.647 \mathrm{eV}$ and the final energy is ${E}_{f} = +0.816\mathrm{ eV}$. The similar results were obtained but with smaller OS for transition at energy gap or higher energies.

**Figure 4:**
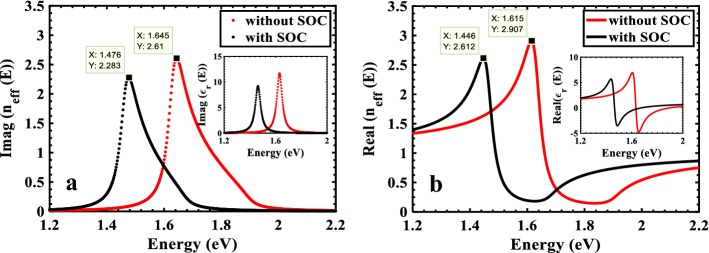
Comparison of the behavior of the (**a**) imaginary and (**b**) real parts of the effective refractive index of the TMDC-PSL with and without SOC. Insets show the real and imaginary parts of the dielectric functions.

There is always some attenuation of light when it goes through a structure. This can be easily explained by defining a complex refractive index, where the imaginary part, ${\mathrm{n}}_{im}$ , represents the extinction coefficient and the real part, ${\mathrm{n}}_{re}$, indicates the phase velocity. The Drude-Lorentz model can be used to determine the structure's effective refractive index and dielectric function since we employ incident light with long wavelengths. The diagram of ${\mathrm{n}}_{im}$ with (without) SOC is shown in Fig. 4a, which represents a Lorentzian peak with half-width of $\Gamma$ at energy gap ${E}_{g}=1.462\mathrm{ eV}$ (${E}_{g}=1.630\mathrm{ eV}$). In other words, at the energy gap the system has a resonance mode and the electronic oscillators excited strongly. The insets in Fig. 4a and b show the variation of dielectric functions of the structure in terms of energy, which equals to the vacuum dielectric function at high energies. so the OS will have the maximum at ${E}_{g}$ (Fig. 3), as in ${\upvarepsilon }_{im}(E)$ and ${n}_{im}(E)$. ${\upvarepsilon }_{re}\left(E\right)=1$ at $E={E}_{g}$(${\upvarepsilon }_{re}\left(E\right)=1$ at $E={E}_{g}$ with a minimum and maximum shifted from ${E}_{g}$ with about $\Gamma$, also the dielectric function of the structure is equal to the vacuum dielectric function at high energies (Fig. 4binset). It is evident from Fig. 4 that the imaginary refractive index varies in the range of $0-2.6$ for radiation energies between 1.2 and 2.2 eV, while the real part is roughly in the range of $0-3$. These values are consistent with the results reported in^39^ for ML-TMDCs. Furthermore, the real and imaginary parts of the refractive index are provided by the Lorentz oscillation model reported for $ML-Mo{Se}_{2}$, which guarantees the validity of these parameters in TMDC-PSL^27^.

**Figure 5:**
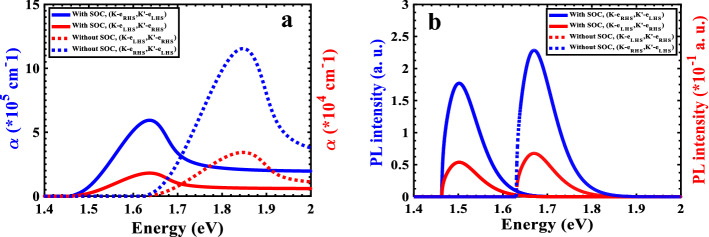
The behavior of (**a**) absorption coefficient (**b**) and photoluminescence intensity for different valleys and polarizations in both cases of with and without SOC.

As discussed before, OS has a maximum value between states ${E}_{i}=-0.8153 eV$ and ${E}_{f}=+0.8153 eV.$ regardless of SOC, we expect the absorption intensity between these two states is maximum. Then, the behavior of the TMDC-PSL absorption coefficient at $K$-valley with up spin and ${\widehat{{\varvec{e}}}}_{{\varvec{R}}{\varvec{H}}{\varvec{S}}}$ polarization, also at ${K}^{\mathrm{^{\prime}}}$-valley with down spin and ${\widehat{{\varvec{e}}}}_{{\varvec{L}}{\varvec{H}}{\varvec{S}}}$ polarization is investigated, as shown in Fig. 5a with dotted blue line. As can be seen, the probability of absorption is zero when the incident light's energy is below the energy gap (${E}_{g}=1.63\mathrm{ eV}$), , and it begins to increase as the incident light's energy approaches the ${E}_{g}$ with the maximum absorption coefficient occurring at the incident energy of $E=1.847\mathrm{ eV}$, having the value of ${\alpha }_{max}=11.54 ({10}^{5} {\mathrm{cm}}^{-1})$. So, the absorption coefficient peak is in the range of visible light spectrum. We then perform this investigation again, this time by taking into account light with ${\widehat{{\varvec{e}}}}_{{\varvec{L}}{\varvec{H}}{\varvec{S}}}$ polarization and ${\widehat{{\varvec{e}}}}_{{\varvec{R}}{\varvec{H}}{\varvec{S}}}$ polarization at the $K$- and ${K}^{\mathrm{^{\prime}}}$-valleys, respectively. The results are depicted in Fig. 5a by the red dashed line. The absorption coefficient peak has a value of ${\alpha }_{max}=0.342 ({10}^{5} {\mathrm{cm}}^{-1})$ at $E=1.847\mathrm{ eV}$. By comparing these two results, it can be seen that the absorption coefficient for the first case is significantly larger than that for second case (33 times), leading to the conclusion that light impinging on the TMDC-PSL with right-circular polarization intends to be coupled by the $K$-valley with an up spin state, whereas light with left-circular polarization intends to be coupled by ${K}^{\mathrm{^{\prime}}}$-valley with a down spin state. Now we study the effect of SOC on absorption coefficient, as it was discovered that the band structure and band gap are significantly affected by taking SOC into account. The optical transition is considered between the initial state ${E}_{i}=-0.6470\mathrm{ eV}$ and the final state ${E}_{f}=+0.8160 eV$, where OS represents a peak and the energy gap size is ${E}_{g}=1.462\mathrm{ eV}$. The blue solid line in Fig. 5a shows the absorption coefficient of the TMDC-PSL, when the radiation light with ${\widehat{{\varvec{e}}}}_{{\varvec{R}}{\varvec{H}}{\varvec{S}}}$ polarization coupled to $K$-valley with up spin as well as the ${\widehat{{\varvec{e}}}}_{{\varvec{L}}{\varvec{H}}{\varvec{S}}}$ polarized light to the ${K}^{\mathrm{^{\prime}}}$-valley with down spin. As can be observed, when the energy of incident light is in close approximity of ${E}_{g}$, the absorption coefficient peak appears at $E=1.637\mathrm{ eV}$ with a value of ${\alpha }_{max}=5.937 \left({10}^{5} {\mathrm{cm}}^{-1}\right).$ It is noteworthy to note that the absorption coefficient peak value for $K$-valley with up spin and incident light of ${\widehat{{\varvec{e}}}}_{{\varvec{L}}{\varvec{H}}{\varvec{S}}}$ polarization, as well as ${K}^{\mathrm{^{\prime}}}$-valley with down spin and incident light of ${\widehat{{\varvec{e}}}}_{{\varvec{R}}{\varvec{H}}{\varvec{S}}}$ polarization are both substantially attenuated with value of ${\alpha }_{max}=0.1804 ({10}^{5} {\mathrm{cm}}^{-1})$, as shown in Fig. 5a with solid red line. Therefore, by taking into account the SOC, it is found that the highest probability of absorption occurs in the TMDC-PSL at $K$-valley with up spin and incident light of ${\widehat{{\varvec{e}}}}_{{\varvec{R}}{\varvec{H}}{\varvec{S}}}$ polarization, as well as the ${K}^{\mathrm{^{\prime}}}$-valley with down spin and incident light of ${\widehat{{\varvec{e}}}}_{{\varvec{L}}{\varvec{H}}{\varvec{S}}}$. The obtained absorption coefficient is significantly higher than that of the GaAs-AlAs conventional vertical superlattices, which is in the order of ${\sim 10}^{4} {\mathrm{cm}}^{-1}$^40^, as well as that of the planar superlattice composed of bilayer-TMDCs, in the order of $\sim {10}^{2} {\mathrm{cm}}^{-1}$ ^15^. Therefore, the TMDC-PSL structure is strongly advised for optical applications.

In Fig. 5b, we study the behavior of photoluminescence intensity (${I}_{PL}$) under different conditions. The trend of the photoluminescence emission spectrum mimics the absorption coefficient $\mathrm{\alpha }$ due to the relation ${I}_{PL}\cong \alpha \left(\mathrm{\hslash }\omega \right){e}^{-(\mathrm{\hslash }\omega -{E}_{g})/{k}_{B}T}$. ${k}_{B}$ is the Boltzmann constant and $T$ is the carrier temperature (almost ambient temperature(. It is obvious that the greater of the separation between the two states ${E}_{i}$ and ${E}_{f}$ (the ground and first excitation states), the greater the probability of radiative recombination is, so the photoluminescence can occur between the two energy states. Therefore, since the energy gap of the TMDC-PSL is large, the highest ${I}_{PL}$ is expected for ${\widehat{{\varvec{e}}}}_{{\varvec{R}}{\varvec{H}}{\varvec{S}}}$ and ${\widehat{{\varvec{e}}}}_{{\varvec{R}}{\varvec{H}}{\varvec{S}}}$ light polarizations at $K$- and ${K}^{\mathrm{^{\prime}}}$-valleys with up and down spins, respectively. The solid and dotted blue lines in Fig. 5b depict the cases with and without SOC, respectively. Since the energy gap is wider in the absence of SOC, the ${I}_{PL}$ is enhanced as previously discussed.

**Figure 6:**
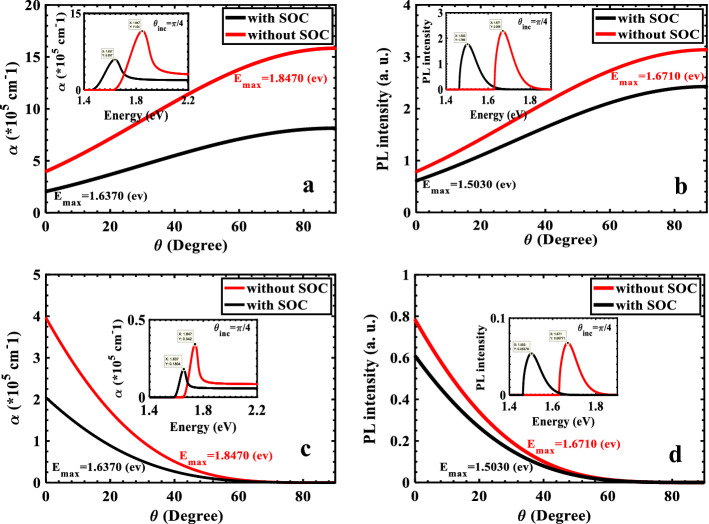
The peak value of (**a**, **c**) the absorption coefficient and (**b**, **d**) the photoluminescence intensity with (black line) and without (red line) SOC versus the incident angle of the radiation light at $K$- and ${K}^{\mathrm{^{\prime}}}$-valleys with up and down spins. The polarization of incident light at $K$- and ${K}^{\mathrm{^{\prime}}}$-valleys are (**a**, **b**) ${\widehat{\mathbf{e}}}_{\mathbf{R}\mathbf{H}\mathbf{S}}$ and ${\widehat{\mathbf{e}}}_{\mathbf{L}\mathbf{H}\mathbf{S}}$ and **(c, d)** ${\widehat{\mathbf{e}}}_{\mathbf{L}\mathbf{H}\mathbf{S}}$ and ${\widehat{\mathbf{e}}}_{\mathbf{R}\mathbf{H}\mathbf{S}}$, respectively. Insets depict for certain value of incident angle as ${\uptheta }_{\mathrm{inc}}=\uppi /4$.

Another important parameter for controlling the optical properties of the TMDC-PSL is the incident angle of the radiation light (${\uptheta }_{inc}$). Here, we study the effect of incident angle on the peak value of the absorption coefficient and that of the photoluminescence intensity in both cases of with and without SOC, as shown in Fig. 6. The insets show the absorption coefficient (Fig. 6a,c) and photoluminescence intensity (Fig. 6b,d) in terms of energy for a particular value of incident angle, $\pi /4$, in both cases of with and without SOC. It can be seen from Fig. 6a and c that the peak value of the absorption coefficient is strongly affected by changing the incident angle of light. In other words, when the incident angle increases the peak value of the optical absorption coefficient at $K$ (${K}^{\mathrm{^{\prime}}}$)-valley with (black line) and without (red line) SOC increases for ${\widehat{e}}_{RHS}$ (${\widehat{e}}_{LHS}$) polarization (Fig. 6a), while it decreases for ${\widehat{e}}_{LHS}$ (${\widehat{e}}_{RHS}$) polarization (Fig. 6c) As a comparison, it is evident from Fig. 6a that, when the incident angle gets $\theta =\pi /2$ (perpendicular to the plane of 2D superlattice) the peak value of the absorption coefficient is three times greater than that of ${\uptheta }_{inc}=0$. The similar behavior is expected for ${I}_{PL}$ due to its direct relation with absorption coefficient, as shown in Fig. 6b and d. It can be seen that the peak value of the ${I}_{PL}$ at $K$ (${K}^{\mathrm{^{\prime}}}$)-valley with (black line) and without (red line) SOC increases for ${\widehat{e}}_{RHS}$ (${\widehat{e}}_{LHS}$) polarization (Fig. 6b), while it decreases for ${\widehat{e}}_{LHS}$ (${\widehat{e}}_{RHS}$) polarization (Fig. 6d). We compare Fig. 6a and c to get more understanding of the impact of incident light angle. Obviously, for very small incident light angles (the incident light wavevector is close to the direction of the periodicity of the 2D structure) the absorption at the $K({K}^{^{\prime}})$-valley experiences almost the same value for both polarizations of the incident light. In contrast, as the incident light wavevector approaches being perpendicular to the 2D plane of the structure, light with right-circular polarization can only be absorbed by $K$-valley and the left-circular polarization can only be absorbed by ${K}^{\mathrm{^{\prime}}}$-valley. As a results, at larger ${\uptheta }_{inc}$ the absorption will be entirely dependent on the polarization. In opto-valleytronic devices, and particularly in optical logic devices, it is possible to accomplish the creation of a zero–one state for light absorption based on the polarization type.

**Figure 7:**
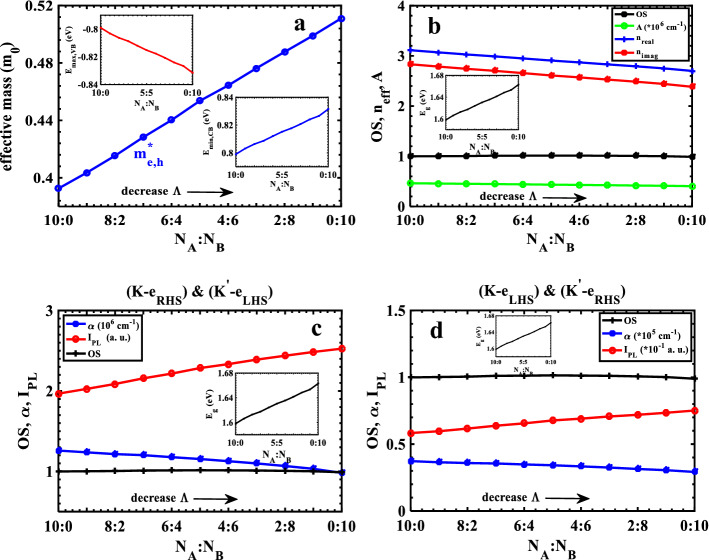
Electronic and optical characteristics of the TMDC-PSL based on the width ratio of the layers without SOC; (**a**) effective mass; (**b**) real (blue) and imaginary (red) effective refractive index, absorbance (green), and OS (black); Absorption coefficient and photoluminescence at $K\left({K}^{^{\prime}}\right)-$ valley with up and down spin for incident light polarizations of (**c**) ${\widehat{{\varvec{e}}}}_{{\varvec{R}}{\varvec{H}}{\varvec{S}}}$ and ${\widehat{{\varvec{e}}}}_{{\varvec{L}}{\varvec{H}}{\varvec{S}}}$ and (**d**) ${\widehat{{\varvec{e}}}}_{{\varvec{L}}{\varvec{H}}{\varvec{S}}}$ , ${\widehat{{\varvec{e}}}}_{{\varvec{R}}{\varvec{H}}{\varvec{S}}}$, respectively.

One of the main objectives in using superlattices is control and engineering of the electronic and optical properties of the structure using the geometrical parameters. One of these parameters is the change in the width ratio of the layers inside the unit cell of the superlattice. It is known that, depending on the synthesis or growth, the M and X atoms are arranged in hexagons in ML-TMDCs^14^. In this study, we define the width of each layer as ${w}_{A(B)}={\mathrm{N}}_{A(B)}{z}_{A(B)}$ to maintain the shape of hexagons along the superlattice. ${\mathrm{N}}_{A(B)}$ is being the number of hexagons and ${z}_{A(B)}$ is lattice constant in layer A(B), as shown in Fig. 1. The width of superlattice unit cell is defined as $\Lambda ={\mathrm{N}}_{A}{z}_{A}+{\mathrm{N}}_{B}{z}_{B}$ and the number of hexagons are fixed at ${\mathrm{N}}_{A}+{\mathrm{N}}_{B}=10$ within the unit cell. Since the width t of the layers $ML-Mo{S}_{2}$ (${z}_{B}=3.193 A$) and $ML-W{Se}_{2}$ (${z}_{A}=3.310 A$) are differ slightly from each other, so as the portion of B layer increases (${\mathrm{N}}_{B}$ increases), the width of the superlattice unit cell becomes smaller. It is obvious from Table. 2 that, from left to right, by increasing (decreasing) the width of the $Mo{S}_{2}$ ($W{Se}_{2}$) layer, the width of the superlattice unit cell, $\Lambda$, varies from $3.3100A$ to $3.1930A$. In according with the band structure for different unit cells, a symmetrical band structure with the same behavior for the valence and conduction bands can be obtained in the absence of SOC. As reported in Table. 2, the maximum valence band and the minimum conduction band shifts to lower (redshift) and higher (blueshift) energies, respectively (Fig. 7a), as a result, we can infer that the energy gap becomes larger as the unit cell size becomes smaller. This behavior is completely consistent with the basic principle that the energy gap is inversely related to the unit cell size in periodic structures (${E}_{g}\propto \frac{1}{\Lambda }$). Since the valence and conduction bands are perfectly symmetric and the bandwidths are the same, the effective mass of electrons and holes in the first conduction and valence bands are equal. These effective masses increases when the portion of the $Mo{S}_{2}$ layer (layer A or B?) ) within the unit cell increase, as shown in Fig. 7a. It is again worth to note that different broadening factors in the system can be indicated. In our calculations, we include these broadening in $\Gamma$ parameter, which causes the energy of the initial and final states to be slightly different from the edge of the valence and conduction bands. This energy difference is approximately equals to $1 meV$ at the edge of each band. The maximum and minimum of the valence and conduction bands varies linearly by reducing $\Lambda$, as depicted in the inset of Fig. 7a. The same behavior is found for OS when the width ratio of the layers within the superlattice unit cell (${N}_{A}:{N}_{B}$) varies (Fig. 7b. Moreover, we investigate the effect of unit cell size, $\Lambda$, on the peak value of the real and imaginary effective refractive indices of the superlattice. As shown in Fig. 7b, the peak value of ${n}_{eff}$ increases linearly as the width of superlattice unit cell decreases, which is a consequence of the linear behavior of the OS. Besides, by decreasing $\Lambda$, the energy gap increases as much as $64\mathrm{ meV}$ (inset of Fig. 7b) . The shift in the peak value of ${n}_{eff}$ towards higher energies is in accordance with the changes in energy gap. The linear behavior of the peak value of the absorbance, which is related to imaginary refractive index as $A=\frac{2{n}_{imag}\omega }{c}$, versus the width ratio of the layers (${N}_{A}:{N}_{B}$) is also depicted in Fig. 7b. As the main objective of this study, we investigate the effect of the structural parameters of the TMDC-PSL on its absorption coefficient. Figure 7c shows the variation of the peak value of the absorption coefficient, as well as the photoluminescence intensity, at $K$(${K}^{\mathrm{^{\prime}}}$)-valley for ${\widehat{e}}_{RHS}$ (${\widehat{e}}_{LHS}$) polarization of the incident light as a function of the width ratio of the constituent layers of the superlattice unit cell. The absorption coefficient of the superlattice decreases linearly by increasing the portion of $Mo{S}_{2}$ layer, , and this leads to an increase in the energy gap, as a result, the shift of absorption threshold towards higher energies. On the other hand, according to Table 2, it is clear that the peak value of the absorption coefficient is constant for all values of $\Lambda$ and has a value of $E=1.847\mathrm{ eV}$. Behavior of the photoluminescence intensity versus ${N}_{A}:{N}_{B}$ is also shown in Fig. 7c, but despite its linear dependence on the absorption coefficient, its peak value increases as the portion of the MoS_2_ layer increases, because the contribution of ${e}^{-(\mathrm{\hslash }\omega -{E}_{g})/{k}_{B}T}$ term is more dominant than the $\alpha \left(\mathrm{\hslash }\omega \right)$. Also, the photoluminescence intensity rises up to $64\mathrm{ meV}$ with an increase in the energy gap. For ${\widehat{e}}_{LHS}$ (${\widehat{e}}_{RHS}$) polarizations of incident light, the same trend is seen at $K$(${K}^{\mathrm{^{\prime}}}$)-valley, but the magnitudes of $\alpha$ and ${I}_{PL}$ are considerably smaller (Fig. 7d). The interesting finding is that the absorbance values confirm the magnitude of the absorption coefficient at $K$(${K}^{\mathrm{^{\prime}}}$)-valley for ${\widehat{e}}_{RHS}$ (${\widehat{e}}_{LHS}$) polarization, which are both factors of $\sim {10}^{6} {\mathrm{cm}}^{-1}$.

**Table 2:** Calculated parameters for the electronic and optical characteristics of the TMDC-PSL based on the width ratio of the layers within the unit cell without SOC.

${N}_{A}:{N}_{B}$	10:0	9:1	8:2	7:3	6:4	5:5	4:6	3:7	2:8	1:9	0:10
$\Lambda (nm)$	3.3100	3.2983	3.2866	3.2749	3.2632	3.2515	3.2398	3.2281	3.2164	3.2047	3.1930
${E}_{max,VB} (eV)$	−0.8000	−0.8031	−0.8061	−0.8091	−0.8120	−0.8150	−0.8180	−0.8211	−0.8241	−08,271	−0.8300
${E}_{min,CB} (eV)$	0.8000	0.8031	0.8061	0.8091	0.8120	0.8150	0.8180	0.8211	0.8241	0.8271	0.8300
${E}_{i} (eV)$	−0.7990	−0.8031	−0.8063	−0.8087	−0.8120	−0.8153	−0.8177	−0.8207	−0.8241	−0.8266	−0.8315
${E}_{f} (eV)$	0.7990	0.8031	0.8063	0.8087	0.8120	0.8153	0.8177	0.8207	0.8241	0.8266	0.8315
${E}_{g} (eV)$	1.6000	1.6062	1.6122	1.6182	1.6240	1.6300	1.6360	1.6422	1.6482	1.6542	1.6600
${m}_{e,1}^{*}$(${m}_{0}$)	0.3926	0.4036	0.4154	0.4283	0.4402	0.4536	0.4643	0.4763	0.4877	0.4990	0.5110
${m}_{h,1}^{*}$(${m}_{0}$)	0.3926	0.4036	0.4154	0.4283	0.4402	0.4536	0.4643	0.4763	0.4877	0.4990	0.5110
$\mathrm{OS}$	0.9792	0.9821	0.9845	0.9908	0.9919	0.9947	0.9925	0.9908	0.9867	0.9816	0.9695
${n}_{real}^{max},{E}_{max}$	3.1130 (1.5830)	3.0700 (1.5910)	3.0280 (1.5970)	2.9900 (1.6030)	2.9490 (1.6090)	2.9070 (1.6150)	2.8700 (1.6210)	2.8300 (1.6270)	2.7920 (1.6310)	2.7510 (1.6370)	2.7000 (1.6470)
${n}_{imag}^{max},{E}_{max}$	2.8330 (1.6130)	2.7870 (1.6210)	2.7420 (1.6270)	2.7020 (1.6310)	2.6570 (1.6370)	2.6100 (1.6450)	2.5720 (1.6490)	2.5280 (1.6550)	2.4860 (1.6590)	2.4410 (1.6670)	2.3840 (1.6770)
$A \left({10}^{5} {cm}^{-1}\right),{E}_{max}$	4.6280 (1.6130)	4.5770 (1.6210)	4.5200 (1.6270)	4.4660 (1.6310)	4.4090 (1.6390)	4.3510 (1.6450)	4.2970 (1.6490)	4.2400 (1.6550)	4.1790 (1.6590)	4.1230 (1.6670)	4.0510 (1.6770)
$\alpha \left({10}^{5} {cm}^{-1}\right),{E}_{max}$ $\left(K,{e}_{RHS}\right),$ $({K}^{^{\prime}},{e}_{LHS})$	12.5900 (1.8470)	12.3700 (1.8470)	12.1500 (1.8470)	12.0200 (1.8470)	11.7700 (1.8470)	11.5400 (1.8470)	11.2700 (1.8470)	10.9900 (1.8470)	10.6700 (1.8470)	10.3200 (1.8470)	9.8130 (1.8470)
$\alpha \left({10}^{5} {cm}^{-1}\right),{E}_{max}$ $\left(K,{e}_{LHS}\right),$ $({K}^{^{\prime}},{e}_{RHS})$	0.3731 (1.8470)	0.3643 (1.8470)	0.3587 (1.8470)	0.3555 (1.8470)	0.3481 (1.8470)	0.3420 (1.8470)	0.3336 (1.8470)	0.3250 (1.8470)	0.3142 (1.8470)	0.3052 (1.8470)	0.2920 (1.8470)
${I}_{PL}\left(a. u.\right),{E}_{max}$ $\left(K,{e}_{RHS}\right),$ $({K}^{^{\prime}},{e}_{LHS})$	1.9670 (1.6390)	2.0260 (1.6470)	2.0870 (1.6530)	2.1570 (1.6570)	2.2160 (1.6650)	2.2850 (1.6710)	2.3340 (1.6750)	2.3900 (1.6810)	2.4380 (1.6850)	2.4850 (1.6930)	2.5260 (1.7030)
${I}_{PL}\left(a. u.\right),{E}_{max}$ $\left(K,{e}_{LHS}\right),$ $({K}^{^{\prime}},{e}_{RHS})$	0.05831 (1.6390)	0.05967 (1.6460)	0.06163 (1.6520)	0.06378 (1.6570)	0.06554 (1.6640)	0.06772 (1.6700)	0.06907 (1.6760)	0.07068 (1.6820)	0.07196 (1.6870)	0.07347 (1.6920)	0.07515 (1.7030)

**Figure 8:**
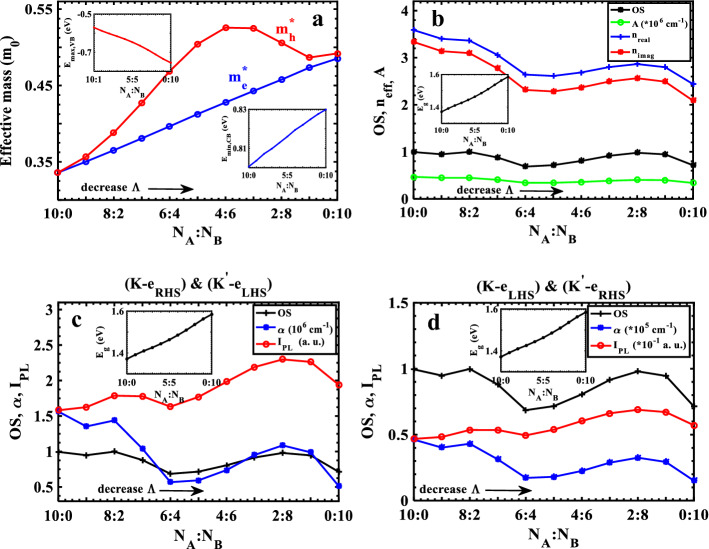
Electronic and optical characteristics of the TMDC-PSL versus the width ratio of the layers with SOC; (**a**) effective mass of carriers; (**b**) real (blue) and imaginary (red) effective refractive index, absorbance (green), and OS (black); Absorption coefficient and photoluminescence at $K\left({K}^{^{\prime}}\right)$-valley with up and down spin for incident light polarizations of (**c**) ${\widehat{{\varvec{e}}}}_{{\varvec{R}}{\varvec{H}}{\varvec{S}}}$ and ${\widehat{{\varvec{e}}}}_{{\varvec{L}}{\varvec{H}}{\varvec{S}}}$ and (**d**) ${\widehat{{\varvec{e}}}}_{{\varvec{L}}{\varvec{H}}{\varvec{S}}}$ , ${\widehat{{\varvec{e}}}}_{{\varvec{R}}{\varvec{H}}{\varvec{S}}}$, respectively.

Finally, we review our previous research on the TMDC-PSL (effective mass, effective refractive index, absorption coefficient and photoluminescence) in the presence of SOC. As was previously mentioned, the primary effect of SOC is the renormalization of the band structure, which has a notable impact on the valence band. In light of SOC, the effective mass of the electron changes less, while that of the hole changes more (Fig. 8a). This modifies the OS results and causes the energy bands' position to change, which immediately affect the behavior of the structure's wavefunctions. By varying the width ratio of layers, $\Lambda ,$ the energy gap can change in magnitude up to $215\mathrm{ meV}$, while it can only change by a maximum of $64meV$ without taking SOC into account. Therefore, other characteristics of the TMDC-PSL properties will be affected by SOC. It should be noticed that the energy gap by value determined by our model superlattice parameters (given in Table 3) are in good agreement with that obtained by the DFT calculations^14^. The energy gap and the peak value of the absorption are greatly affected by increasing the portion of the $Mo{S}_{2}$ layer. In the presence of SOC at $K$(${K}^{\mathrm{^{\prime}}}$)-valley for ${\widehat{e}}_{RHS}$ (${\widehat{e}}_{LHS}$) polarizations of the incident light, Fig. 8b shows the behavior of ${n}_{eff}$ and $A$. Figure 8c and d show the behavior of $\alpha$ and ${I}_{PL}$ versus the width ratio of layers within the superlattice unit cell at $K\left({K}^{^{\prime}}\right)$-valley with up and down spin for incident light polarizations of ${\widehat{{\varvec{e}}}}_{{\varvec{R}}{\varvec{H}}{\varvec{S}}}$ , ${\widehat{{\varvec{e}}}}_{{\varvec{L}}{\varvec{H}}{\varvec{S}}}$ and ${\widehat{{\varvec{e}}}}_{{\varvec{L}}{\varvec{H}}{\varvec{S}}}$ , ${\widehat{{\varvec{e}}}}_{{\varvec{R}}{\varvec{H}}{\varvec{S}}}$, respectively. The behavior of the OS has an impact on these superlattice properties. It is clear that there is an oscillatory tendency in these parameters when compared to those shown in Fig. 7, which is utilized to engineer the optical and transport properties of TMDC-PSL to implement in optical devices.

**Table 3:** Parameters of the electronic and optical characteristics of the TMDC-PSL versus the width ratio of the layers with SOC.

${N}_{A}:{N}_{B}$	10:0	9:1	8:2	7:3	6:4	5:5	4:6	3:7	2:8	1:9	0:10
$\Lambda (nm)$	3.3100	3.2983	3.2866	3.2749	3.2632	3.2515	3.2398	3.2281	3.2164	3.2047	3.1930
${E}_{max,VB} (eV)$	−0.5700	−0.5875	−0.6022	−0.6162	−0.6308	−0.6470	−0.6656	−0.6871	−0.7110	−0.7349	−0.7550
${E}_{min,CB} (eV)$	0.8000	0.8032	0.8062	0.8091	0.8121	0.8151	0.8181	0.8211	0.8241	0.8271	0.8300
${E}_{i} (eV)$	−0.5706	−0.5878	−0.6027	−0.6162	−0.6308	−0.6470	−0.6659	−0.6871	−0.7110	−0.7350	−0.7550
${E}_{f} (eV)$	0.8006	0.8037	0.8072	0.8098	0.8129	0.8160	0.8194	0.8220	0.8249	0.8276	0.8299
${E}_{g} (eV)$	1.3700	1.3907	1.4084	1.4253	1.4429	1.4621	1.4837	1.5082	1.5351	1.5620	1.5850
${E}_{g} \left(eV\right),DFT$	–	–	1.41	1.42	1.41	–	–	–	–	–	–
${m}_{e,1}^{*}$(${m}_{0}$)	0.3359	0.3502	0.3652	0.3807	0.3965	0.4126	0.4278	0.4428	0.4576	0.4734	0.4853
${m}_{h,1}^{*}$(${m}_{0}$)	0.3359	0.3565	0.3882	0.4271	0.4687	0.5043	0.5257	0.5250	0.5059	0.4866	0.4920
$OS$	0.9949	0.9478	0.9981	0.8799	0.6857	0.7163	0.8059	0.9153	0.9794	0.9454	0.7139
${n}_{real}^{max},{E}_{max}$	3.5880 (1.3560)	3.4020 (1.3760)	3.3640 (1.3940)	3.0600 (1.4100)	2.6410 (1.4280)	2.6120 (1.4460)	2.6870 (1.4700)	2.8010 (1.4920)	2.8650 (1.5210)	2.7990 (1.5470)	2.4390 (1.5690)
${n}_{imag}^{max},{E}_{max}$	3.3360 (1.3840)	3.1390 (1.4040)	3.0970 (1.4220)	2.7730 (1.4380)	2.3150 (1.4560)	2.2830 (1.4760)	2.3670 (1.4980)	2.4940 (1.5230)	2.5640 (1.5490)	2.4910 (1.5750)	2.0870 (1.5970)
$A \left({10}^{5} {cm}^{-1}\right),{E}_{max}$	4.6810 (1.3840)	4.4700 (1.4040)	4.4710 (1.4220)	4.0470 (1.4380)	3.4170 (1.4560)	3.4160 (1.4760)	3.5950 (1.4980)	3.8480 (1.5230)	4.0240 (1.5490)	3.9810 (1.5770)	3.3810 (1.5970)
$\alpha \left({10}^{5} {cm}^{-1}\right),{E}_{max}$ $\left(K,{e}_{RHS}\right),$ $({K}^{^{\prime}},{e}_{LHS})$	15.6400 (1.6890)	13.5300 (1.6810)	14.3800 (1.6930)	10.3900 (1.6630)	5.6790 (1.6210)	5.9370 (1.6370)	7.3860 (1.6690)	9.5130 (1.7090)	10.8600 (1.7450)	9.8810 (1.7630)	5.1370 (1.7370)
$\alpha \left({10}^{5} {cm}^{-1}\right),{E}_{max}$ $\left(K,{e}_{LHS}\right),$ $({K}^{^{\prime}},{e}_{RHS})$	0.4625 (1.6890)	0.4017 (1.6810)	0.4311 (1.6930)	0.3125 (1.6630)	0.1721 (1.6210)	0.1804 (1.6370)	0.2250 (1.6690)	0.2875 (1.7090)	0.3253 (1.7450)	0.2929 (1.7630)	0.1512 (1.7370)
${I}_{PL}\left(a. u.\right),{E}_{max}$ $\left(K,{e}_{RHS}\right),$ $({K}^{^{\prime}},{e}_{LHS})$	1.5810 (1.4120)	1.6250 (1.4320)	1.7870 (1.4500)	1.7780 (1.4660)	1.6320 (1.4820)	1.7690 (1.5030)	1.9820 (1.5250)	2.1900 (1.5490)	2.2980 (1.5750)	2.2630 (1.6030)	1.9410 (1.6230)
${I}_{PL}\left(a. u.\right),{E}_{max}$ $\left(K,{e}_{LHS}\right),$ $({K}^{^{\prime}},{e}_{RHS})$	0.04678 (1.4120)	0.04823 (1.4320)	0.05356 (1.4500)	0.05348 (1.4660)	0.04946 (1.4820)	0.05378 (1.5030)	0.06037 (1.5250)	0.06621 (1.5490)	0.06885 (1.5750)	0.06709 (1.6030)	0.05715 (1.6230)

## Conclusions

In conclusion, we studied the electronic band structure and optical absorption in a planar TMDC superlattices containing alternatively arranged MoS_2_ and WSe_2_. It is observed that by considering the SOC effect, the valence band experiences a few hundred meV blueshift while the conduction band will have slight change. Due to the parabolic band structure at $\mathrm{K}=0$, we were able to calculate the effective mass for different bands using the effective mass approximation (with SOC effect) as ${m}_{e,1}^{*}=0.4126 {m}_{0}$ and ${m}_{h,1}^{*}=0.5043 {m}_{0}$. By investigating the oscillator strength behavior, we found strong optical absorption at the edge of the conduction band in the interband band transition. Within Drude-Lorentz model we obtained the effective refractive index of the structure so that for the energy range of $\mathrm{E}=1.2-2.2\mathrm{ eV}$, the effective refractive index will be in the range of ${n}_{re}=0-3$ and ${n}_{im}=0-2.6$. By studying the optical characteristics of the PSL-TMDC, we observed that for the states (${\varvec{S}}\uparrow , {\varvec{K}}, {\widehat{{\varvec{e}}}}_{{\varvec{R}}{\varvec{H}}{\varvec{S}}}$) and (${\varvec{S}}\downarrow , {{\varvec{K}}}^{\mathrm{^{\prime}}}, {\widehat{{\varvec{e}}}}_{{\varvec{L}}{\varvec{H}}{\varvec{S}}}$) with SOC, the absorption coefficient starts to be decreased from ${\alpha }_{max}=11.54 ({10}^{5} {\mathrm{cm}}^{-1})$ to ${\alpha }_{max}=5.937 ({10}^{5} {\mathrm{cm}}^{-1})$ and also by changing the polarization of the above states, i.e. for the states (${\varvec{S}}\uparrow , {\varvec{K}}, {\widehat{{\varvec{e}}}}_{{\varvec{L}}{\varvec{H}}{\varvec{S}}}$) and (${\varvec{S}}\downarrow , {{\varvec{K}}}^{\mathrm{^{\prime}}}, {\widehat{{\varvec{e}}}}_{{\varvec{R}}{\varvec{H}}{\varvec{S}}}$) the absorption coefficient will be equal to ${\alpha }_{max}=0.1804 ({10}^{5} {\mathrm{cm}}^{-1})$, in the other words, it will be reduced 33 times. Due to the variation in the behavior of the absorption coefficient, by applying the effects of SOC in one hand and the change in the polarization of the incident light in the other hand, almost the same behavior is observed for the photoluminescence intensity of the structure. One of the important parameters to control the optical absorption of the structure is the incident angle of light. When the light propagation direction is in the direction of the periodicity of the system (${\uptheta }_{inc}=0$), the absorption by the ${\varvec{K}}$(${{\varvec{K}}}^{\mathrm{^{\prime}}}$) valley experiences almost the same amount for both right and left circular polarizations. But as the light propagation direction becomes close to the perpendicular to the 2D plane of the structure (${\uptheta }_{inc}>0$) light with right circular polarization will be absorbed only by the ${\varvec{K}}$ valley and left circular polarization by the ${{\varvec{K}}}^{\mathrm{^{\prime}}}$ valley. Our theoretical approach paves the way towards designing optovalleytronics devices in which the optical properties with electronic structure can be adjusted and controlled with considerable value of precise by the means of superlattice structure.

## Data Availability

The datasets used and/or analyzed during the current study are available from the corresponding author on reasonable request.
